# A susceptibility locus in the *IL12B* but not *LILRA3* region is associated with vascular damage in Takayasu arteritis

**DOI:** 10.1038/s41598-021-93213-9

**Published:** 2021-07-01

**Authors:** Keiichiro Kadoba, Ryu Watanabe, Takeshi Iwasaki, Toshiki Nakajima, Koji Kitagori, Shuji Akizuki, Kosaku Murakami, Ran Nakashima, Motomu Hashimoto, Masao Tanaka, Koichiro Ohmura, Akio Morinobu, Chikashi Terao, Hajime Yoshifuji

**Affiliations:** 1 Department of Rheumatology and Clinical Immunology, Graduate School of Medicine, Kyoto University, Kyoto, Japan; 2 Department of Advanced Medicine for Rheumatic Diseases, Graduate School of Medicine, Kyoto University, 54 Shogoin-Kawahara-cho, Sakyo-ku, Kyoto, 606-8507 Japan; 3 Center for Genomic Medicine, Graduate School of Medicine, Kyoto University, Kyoto, Japan; 4 Department of Clinical Immunology and Rheumatology, The Tazuke-Kofukai Medical Research Institute, Kitano Hospital, Osaka, Japan; 5 Laboratory for Statistical and Translational Genetics, Center for Integrative Medical Sciences, RIKEN Center for Integrative Medical Sciences, Yokohama, Japan; 6 Clinical Research Center, Shizuoka General Hospital, Shizuoka, Japan; 7 The Department of Applied Genetics, The School of Pharmaceutical Sciences, University of Shizuoka, Shizuoka, Japan

**Keywords:** Immunology, Rheumatology

## Abstract

HLA-B*52 is an established genetic factor in Takayasu arteritis (TAK). Recently, single nucleotide polymorphisms (SNPs) in *IL12B* (rs6871626) and *LILRA3* (rs103294) were newly identified as non-HLA susceptibility loci in TAK. Here, we examined how these SNPs contribute to clinical characteristics and vascular damage in TAK. We retrospectively reviewed the medical records of 99 TAK patients enrolled in our previous genome-wide association study, and whose genotypes for *IL12B* rs6871626, *LILRA3* rs103294, and HLA-B*52 were available. Incidence of aortic regurgitation (AR) was significantly associated with the A allele (risk allele) of *IL12B* rs6871626 (CC 42%, AC 61%, AA 81%; p = 0.0052; odds ratio [OR] 2.45), as well as with the incidence of hypertension (p = 0.049; OR 1.82) and the proportion of patients who underwent aortic valve replacement (p = 0.023; OR 3.64). Regarding vascular damage, there was positive correlation between the Takayasu Arteritis Damage Score and the A allele of *IL12B* rs6871626 (CC 3.42 ± 2.71, AC 4.06 ± 3.25, AA 6.00 ± 2.81; p = 0.0035; β = 1.35) and between the Vasculitis Damage Index and the A allele (CC 3.47 ± 1.98, AC 4.33 ± 2.40, AA 5.37 ± 2.22; p = 0.0054; β = 0.96). Contrarily, no correlation was found between *LILRA3* rs103294 and vascular damage. In the present study, *IL12B* rs6871626 was associated with vascular damage in TAK, whereas *LILRA3* rs103294 was not. Genotyping of *IL12B* rs6871626 may help to identify patients at risk of disease progression.

## Introduction

Takayasu arteritis (TAK) is an idiopathic, chronic, inflammatory disease, characterized by granulomatous panarteritis of the aorta and its major branches, and typically presents before the age of 40 years^1^. TAK is a potentially devastating disease, often causing substantial vascular damage in affected patients. Stenoses and occlusions of major aortic branches can result in serious ischemic complications such as cerebral infarction, visual loss, and claudication. In addition, patients can develop rapidly expanding aneurysms resulting in aortic rupture. Cardiac complications are common, and 30–60% of patients develop aortic regurgitation (AR)^1,2^.

HLA-B*52 is a well-established genetic component associated with TAK worldwide^3^. As a non-HLA susceptibility locus of TAK, we previously reported a single nucleotide polymorphism (SNP), rs6871626, in the *IL12B* region^4,5^. *IL12B* rs6871626 was positively associated with AR and inflammatory marker levels^4^. In addition, Matsumura et al. identified the association between *IL12B* rs6871626 and disease severity defined by early onset, steroid resistance, and/or relapsing disease course^6^. However, it has not been elucidated whether *IL12B* rs6871626 is associated with vascular damage. In addition to *IL12B* rs6871626, we recently identified four unreported susceptibility loci for TAK^5^. Among these, rs103294 in the *LILRA3* region showed a significant epistasis effect with HLA-B*52^5^. *LILRA3* belongs to the LILR family expressed on various leukocytes, and its detailed functions and mechanisms are still not known^5,7,8^. The association between *LILRA3* rs103294 and clinical characteristics in TAK, including vascular damage, has never been examined.

Damage to systemic vasculitides is defined as irreversible scars in the involved organs, which may be caused by either disease activity or therapeutic approaches^9^. The damage measures for TAK, such as the Takayasu arteritis damage score (TADS)^10^ and Vasculitis Damage Index (VDI) have been developed^11^. The former is a TAK-specific damage measure focusing mainly on disease-related damage in the cardiovascular system, whereas the latter is a validated damage score for use in all types of systemic vasculitides, which also captures treatment-related damage well^9,12^. In addition, as an imaging-based scoring system to evaluate systemic angiographic damages in large vessel vasculitis, Arteritis Stenosis Score (ASS), Arteritis Dilation Score (ADS), and Arteritis Composite Score (ACS) have been developed and validated^13^. ASS and ACS were reported to correlate with TADS and reflect vascular damage rather than disease activity. These are novel vascular outcome measures with wide applicability and utility for clinical trials.

Here, we evaluated the association of *IL12B* rs6871626 and *LILRA3* rs103294 with vascular damage and other clinical characteristics of TAK.

## Results

### Demographic characteristics and organ involvements

**Figure 1:**
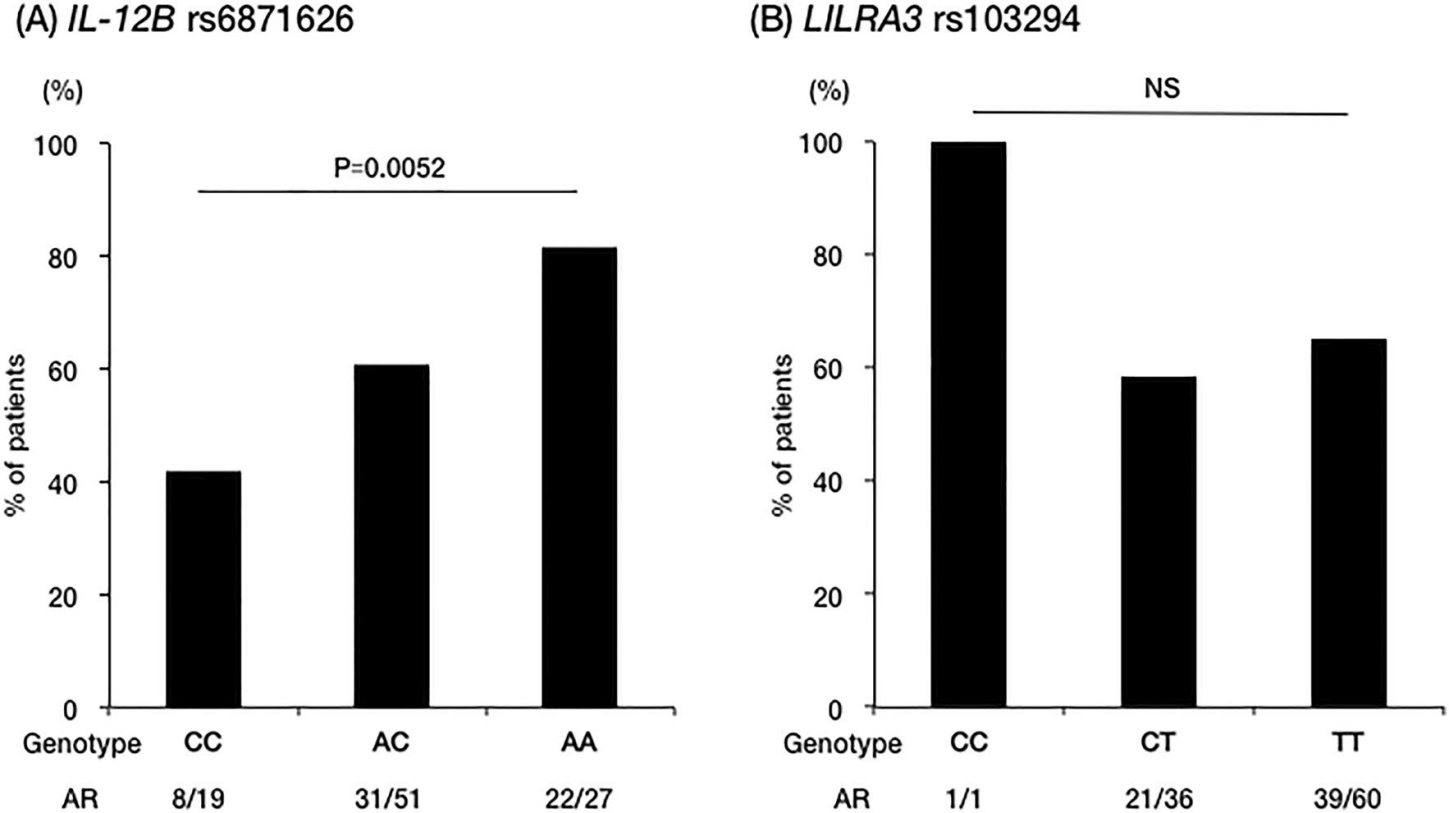
Association of genotypes with the frequency of AR. The association of (**A**) *IL12B* rs6871626 and (**B**) *LILRA3* rs103294 with the frequency of AR. *AR* aortic regurgitation, *NS* not significant.

A summary of demographic characteristics and organ involvements stratified by genotypes of *IL12B* rs6871626 and *LILRA3* rs103294 genotypes are shown in Table 1. Age at onset and male–female ratio were not associated with the A allele (risk allele) of *IL12* rs6871626; however, the incidence of AR was positively associated with this allele in the additive model (CC 8/19 [42%], AC 31/51 [61%], AA 22/27 [81%]; p = 0.0052; OR 2.45, 95% CI 1.27–4.73). No association was found between the prevalence of AR and the genotypes of *LILRA3* rs103294 (Fig. 1). The incidence of hypertension was also associated with the A allele of *IL12B* rs6871626 in the additive model (CC 10/19 [53%], AC 23/53 [45%], AA 21/27 [78%]; p = 0.049; OR 1.82; 95% CI 0.99–3.36); however, no association was found between the prevalence of hypertension and genotypes of *LILRA3* rs103294. Other organ involvements were not associated with either *IL12B* rs6871626 or *LILRA3* rs103294.

**Table 1:** Backgrounds and organ involvements of the subjects.

	Total	*IL-12B* rs6871626				*LILRA3* rs103294			
		CC	AC	AA	p value	CC	CT	TT	p value
Number	99	19	53	27		1	38	60	
Age at onset (years)	31 ± 15	33 ± 16	30 ± 14	32 ± 15	0.91	45	35 ± 17	29 ± 13	0.052
Female	94 (95%)	18 (95%)	53 (100%)	23 (85%)	0.067	1 (100%)	36 (95%)	57 (95%)	0.99
Visual loss	3 (3%)	0 (0%)	2 (4%)	1 (4%)	0.51	0 (0%)	1 (3%)	2 (3%)	0.84
Hypertension	54 (56%)	10 (53%)	23 (45%)	21 (78%)	0.049*	1 (100%)	18 (50%)	35 (58%)	0.64
Ischemic heart disease	6 (6%)	1 (5%)	4 (8%)	1 (4%)	0.76	0 (0%)	1 (3%)	5 (8%)	0.23
Cerebrovascular event	9 (9%)	0 (0%)	5 (10%)	4 (15%)	0.088	0 (0%)	3 (8%)	6 (10%)	0.71
Renal replacement therapy	1 (1%)	0 (0%)	1 (2%)	0 (0%)	0.9	0 (0%)	0 (0%0	1 (2%)	0.33
Inflammatory bowel disease	7 (7%)	1 (5%)	2 (4%)	4 (15%)	0.16	0 (0%)	2 (6%)	5 (8%)	0.56
Ulcerative colitis	5 (5%)	1 (5%)	1 (2%)	3 (11%)	0.28	0 (0%)	2 (6%)	3 (5%)	0.97
Crohn disease	2 (2%)	0 (0%)	1 (2%)	1 (4%)	0.37	0 (0%)	0 (0%)	2 (3%)	0.16

Data are n (%) or mean ± standard deviation.

In the dominant model, we did not observe significant associations between *IL12B* rs6871626 and AR or hypertension, although the incidence tended to be higher in risk genotypes of *IL12B* rs6872626 (Supplementary Table).

### Treatment profiles

Treatment profiles stratified by genotypes of *IL12B* rs6871626 and *LILRA3* rs103294 are summarized in Table 2. No association was found between *IL12B* rs6871626 or *LILRA3* rs103294 and the use of glucocorticoids, oral immunosuppressants, or biologics, although the proportion of biologic users tended to be higher in the risk genotypes of *IL12B* rs6871626.

**Table 2:** Treatment profiles of the subjects.

	Total	*IL-12B* rs6871626				*LILRA3* rs103294			
		CC	AC	AA	p value	CC	CT	TT	p value
Number		19	53	27		1	38	60	
**Medication**									
Glucocorticoid	79 (80%)	15 (79%)	42 (79%)	22 (81%)	0.82	1 (100%)	30 (79%)	48 (80%)	0.97
Oral immunosuppressant	38 (39%)	5 (26%)	22 (42%)	11 (41%)	0.38	1 (100%)	14 (38%)	23 (38%)	0.72
Biologic	17 (17%)	1 (5%)	10 (19%)	6 (22%)	0.15	1 (100%)	6 (16%)	10 (17%)	0.52
Surgery or catheterization	34 (35%)	8 (42%)	12 (23%)	14 (52%)	0.31	0 (0%)	11 (30%)	23 (38%)	0.29
CABG	5 (5%)	2 (11%)	3 (6%)	0 (0%)	0.1	0 (0%)	1 (3%)	4 (7%)	0.35
PCI	7 (7%)	2 (11%)	4 (8%)	1 (4%)	0.37	0 (0%)	1 (3%)	6 (10%)	0.14
Aortic valve replacement	9 (9%)	1 (5%)	2 (4%)	6 (22%)	0.023*	0 (0%)	4 (11%)	5 (8%)	0.78
Aneurysm repair	10 (10%)	2 (11%)	2 (4%)	6 (22%)	0.11	0 (0%)	6 (16%)	4 (7%)	0.2
Bypass surgery	9 (9%)	2 (11%)	3 (6%)	4 (15%)	0.51	0 (0%)	2 (5%)	7 (12%)	0.26

*CABG* coronary artery bypass grafting, *PCI* percutaneous coronary intervention.

Data are n (%) or mean ± standard deviation.

Regarding surgery or catheter intervention, the proportion of patients who had undergone aortic valve replacement was associated with the A allele of *IL12B* rs6871626 (CC 1/19 [5%], AC 2/53 [4%], AA 6/27 [22%]; p = 0.023; OR 3.64, 95% CI 1.08–12.24), whereas no association was found with genotypes of *LILRA3* rs103294. The overall proportion of surgery or catheter interventions and the proportion of patients who had undergone other specific vascular interventions, such as coronary artery bypass graft and percutaneous coronary intervention, were not associated with either *IL12B* rs6871626 or *LILRA3* rs103294.

### Arterial involvement

**Figure 2:**
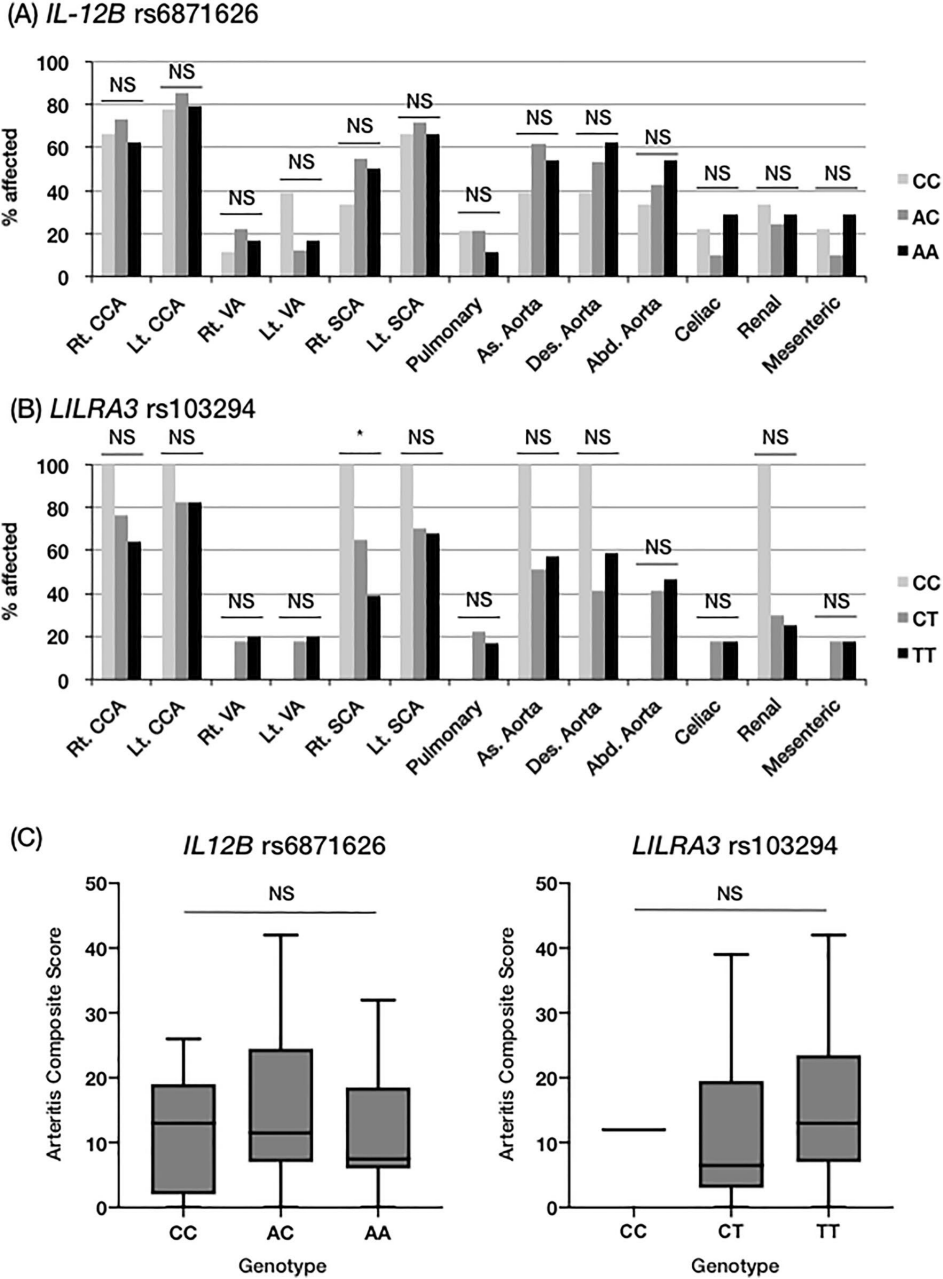
Association of genotypes with arterial involvement. (**A,B**) The association of *IL12B* rs6871626 and *LILRA3* rs103294 with the distribution of arterial involvement. (**C**) The association of *IL12B* rs6871626 and *LILRA3* rs103294 with Arteritis Composite Score. *Rt* right, *Lt* left, *As* ascending, *Des* thoracic descending, *Abd* abdominal descending, *NS* not significant. *P < 0.05.

Figure 2A,B summarizes the distribution of arterial involvement. No association was found between arterial involvement and genotypes of *IL12B* rs6871626 or *LILRA3* rs103294, except for a lower incidence of right subclavian artery involvement in patients with the T allele (risk allele) of *LILRA3* rs103294 (CC 1/1 [100%], CT 22/34 [65%], TT 22/56 [39%]; p = 0.010; OR 0.36, 95% CI 0.14–0.79) (Fig. 2A,B).

To quantify systemic vascular damage on imaging, we calculated ASS, ADS, and ACS. We did not observe the association between these scores and genotypes of *IL12B* rs6871626 or *LILRA3* rs103294 (Fig. 2C, Supplementary Figure).

### Vasculitis-associated damage measures

**Figure 3:**
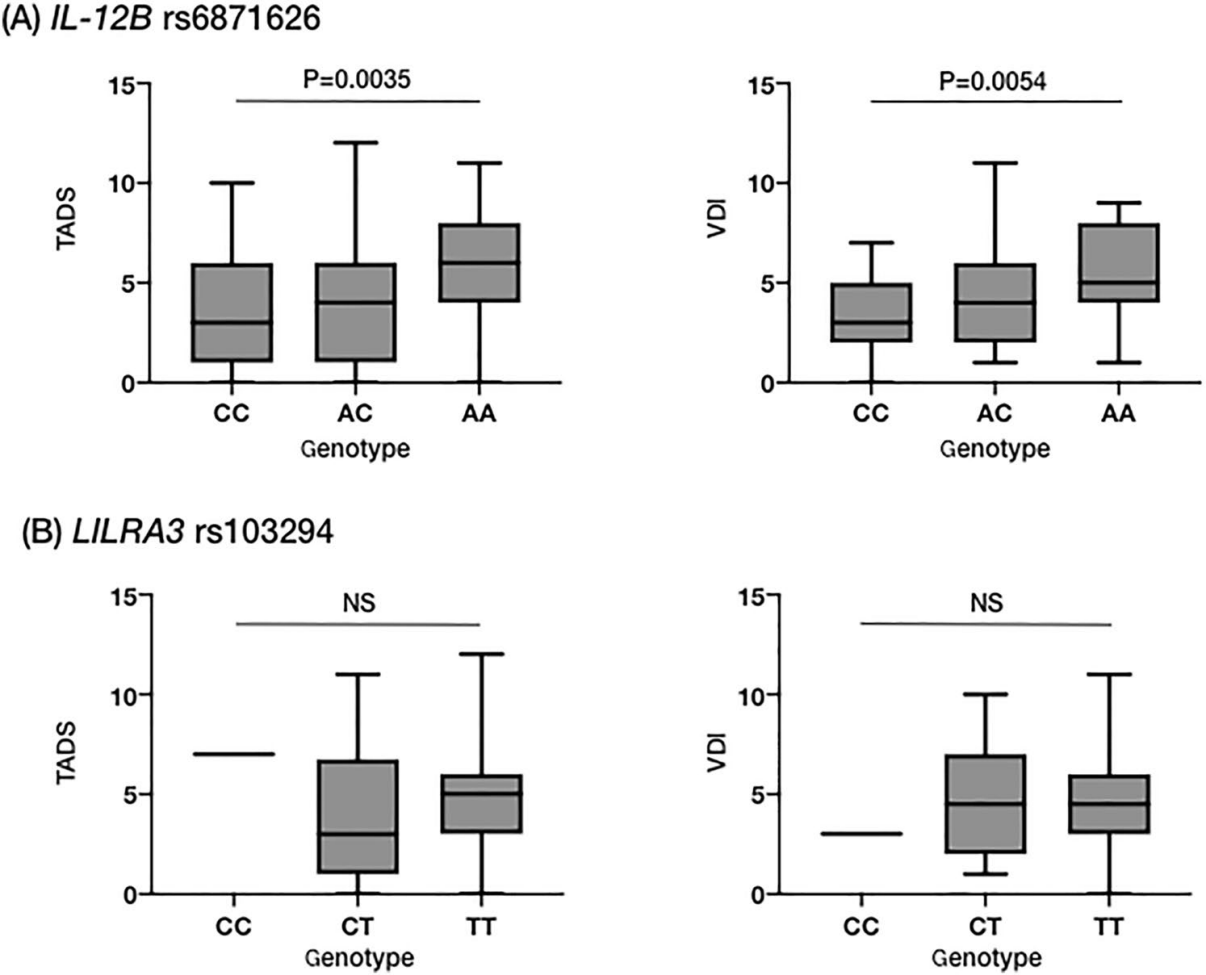
Association of genotypes with vasculitis-associated damage measures. The association of (**A**) *IL12B* rs6871626 and (**B**) *LILRA3* rs103294 with TADS and VDI. *TADS* Takayasu arteritis damage score, *VDI* Vasculitis Damage Index, *NS* not significant.

Then, we evaluated vasculitis-associated damage measures using TADS and VDI. There was a positive correlation between TADS and the A allele of *IL12B* rs6871626 in the additive model (CC 3.42 ± 2.71, AC 4.06 ± 3.25, AA 6.00 ± 2.81; p = 0.0035; β = 1.35) (Fig. 3A). In addition, VDI was positively correlated with the A allele in the additive model (CC 3.47 ± 1.98, AC 4.33 ± 2.40, AA 5.37 ± 2.22; p = 0.0054; β = 0.96) (Fig. 3A). When adjusted for disease duration, the correlation of TADS and VDI with the A allele of *IL12B* rs6871626 remained statistically significant (p = 0.013, β = 1.11 for TADS; p = 0.025, β = 0.72 for VDI). No correlation was found between *LILRA3* rs103294 and TADS or VDI (Fig. 3B).

In the dominant model, we did not observe significant associations between *IL12B* rs6871626 and TADS or VDI, although their scores tended to be higher in risk genotypes of *IL12B* rs6872626 (Supplementary Table).

### Association between clinical characteristics and LILRA3 rs103294 genotypes in HLA-B*52-positive patients

We previously reported a significant epistasis effect between HLA-B*52 and *LILRA3* rs103294^5^. The TT genotype with HLA-B*52 was associated with TAK susceptibility, whereas the TT genotype without HLA-B*52 was not. In other words, *LILRA3* rs103294 functions as a TAK susceptibility gene only in the presence of HLA-B*52. Therefore, we hypothesized that the association between clinical characteristics and *LILRA3* rs103294 is found only in HLA-B*52-positive patients. However, as summarized in Table 3, there was no significant association between clinical characteristics and *LILRA3* rs103294 even in HLA-B*52-positive patients. In addition, the association between clinical characteristics and *LILRA3* rs103294 was not observed in HLA-B*52-negative patients (data not shown).

**Table 3:** Clinical characteristics of HLA-B52-positive patients stratified by LILRA3 genotypes.

	Total	*LILRA3* rs103294			
		CC	CT	TT	p value
Number		1	16	39	
Age at onset (years)	32 ± 13	45	34 ± 12	31 ± 13	0.27
Female	53 (95%)	1 (100%)	16 (100%)	36 (92%)	0.13
Visual loss	1 (2%)	0 (0%)	0 (0%)	1 (3%)	0.4
Hypertension	31 (56%)	1 (100%)	8 (53%)	22 (56%)	0.82
Aortic regurgitation	39 (71%)	1 (100%)	11 (73%)	27 (69%)	0.57
Ischemic heart disease	3 (5%)	0 (0%)	0 (0%)	3 (8%)	0.14
Cerebrovascular accident	5 (9%)	0 (0%)	1 (7%)	4 (10%)	0.59
Pulmonary artery involvement	14 (25%)	0 (0%)	6 (40%)	8 (21%)	0.31
Renal artery involvement	12 (22%)	1 (100%)	4 (27%)	7 (18%)	0.15
TADS	4.3 ± 3.0	7	3.7 ± 3.3	4.5 ± 3.0	0.78
VDI	4.2 ± 2.4	3	4.6 ± 2.9	4.1 ± 2.2	0.71
**Medication**					
Oral immunosuppressant	26 (46%)	1 (100%)	7 (44%)	18 (46%)	0.73
Biologic	11 (20%)	1 (100%)	3 (19%)	7 (18%)	0.34
Surgery or catheter intervention	17 (30%)	0 (0%)	4 (25%)	13 (33%)	0.39

*TADS* Takayasu arteritis damage score, *VDI* vasculitis damage index.

Data are n (%) or mean ± standard deviation.

## Discussion

In the present study, we examined for the first time the impact of *IL12B* rs6871626 and *LILRA3* rs103294, which were newly identified susceptibility loci, on clinical characteristics and vascular damage in TAK patients. Our study demonstrated that *IL12B* rs6871626, but not *LILRA3* rs103294, was associated with organ involvement and vascular damage assessed by TADS and VDI. Our study results suggest that patients harboring risk alleles of *IL12B* rs6871626 may be at risk of disease progression, requiring close monitoring. In addition, we detected for the first time an association between *IL12B* rs6871626 and hypertension in TAK, which was not attributed to renal artery involvement. Low compliance of arterial walls in patients with risk alleles may lead to systemic hypertension.

Our results are compatible with previous reports showing the association of *IL12B* rs6871626 with AR, inflammatory marker levels, and disease severity^4,6^. *IL12B* encodes IL-12p40, a common subunit of IL-12 and IL-23^14^. More specifically, IL-12p40-dependent cytokines include IL-12p70, IL-12p80, and IL-23^15^. IL-12p70 is composed of IL-12p40 and IL-12p35 subunits, while IL-12p80 is a homodimer of IL-12p40 and considered as an antagonist of IL-12p70 function^15^. Although there have been conflicting results regarding whether IL-12p70^16–19^ and IL-23^19–22^ participate in the pathogenesis of TAK, our recent work has demonstrated that both IL-12p40 and IL-12p70 were enriched in the plasma of TAK patients, and was more pronounced in patients with the risk allele of *IL12B* rs6871626^23^. Taken together, the risk genotype of *IL12B* rs6871626 may contribute to the severe phenotype of TAK through subsequent overproduction of IL-12p40 and IL-12p70.

This hypothesis is supported by our pilot study using ustekinumab (UST), a monoclonal antibody targeting IL-12p40, for treating TAK. We confirmed the improvement of symptoms, inflammatory marker levels, and safety in the first three months of treatment with UST^24^. We have also conducted an observational study evaluating long-term outcomes of refractory TAK patients treated with biologics, and found that UST has effects on reducing inflammatory marker levels and glucocorticoid dose^25^. *IL12B* rs6871626 has also been reported as a susceptibility locus in inflammatory bowel disease^26,27^ and ankylosing spondylitis^28^. Co-occurrence of TAK and ulcerative colitis is not uncommon, and the two diseases are reported to share a significant proportion of genetic overlap^29,30^. IL-12p40 blockade is an approved treatment of choice for inflammatory bowel disease^31,32^. In light of this, further studies are warranted to assess the clinical efficacy of IL-12p40 blockade in TAK.

In this study, we did not identify any influence of *LILRA3* rs103294 on clinical phenotypes of TAK, except for a lower incidence of right subclavian artery involvement in the risk genotype. In our study population, the genotype of *LILRA3* rs103294 was strongly biased towards risk genotypes, with only one patient having the CC genotype. This may have limited the power of this study to identify the association of *LILRA3* rs103294 with clinical characteristics of TAK. Another possible explanation is that *LILRA3* rs103294 only affects disease susceptibility and is irrelevant to disease progression, thus contributing differently to TAK pathogenesis from *IL12B* rs6871626. It is unclear why patients with the non-risk allele showed a significantly higher frequency of right subclavian artery lesions (Fig. 2), but this may have been confounded by the systemically advanced vascular lesions of only patients with the CC genotype.

LILRA3 is the only soluble form of LILR and is not detected on the cell surface^7^. It is speculated that LILRA3 functions as an antagonist or a negative regulator of other LILRs, but its detailed functions and mechanisms are still not known^7,8^. The risk allele of *LILRA3* rs103294 is associated with decreased expression of LILRA3^5^. Therefore, decreased levels of LILRA3 may contribute to the pathogenesis of TAK. To date, associations between LILRA3 and various autoimmune diseases have been reported. LILRA3 deficiency is a risk factor for multiple sclerosis in German and Spanish populations^33,34^. Homozygous LILRA3 deletion is associated with Sjögren’s syndrome in a German population^35^, while functional LILRA3 is a susceptibility factor for Sjögren’s syndrome in a Chinese population^36^. In addition, functional (non-deleted) LILRA3 is a susceptibility factor for systemic lupus erythematosus and rheumatoid arthritis, especially for anti-citrullinated protein antibody-positive cases^36,37^. Considering these previous reports, it can be speculated that LILRA3 contributes differently to the pathogenesis of different autoimmune diseases. Further study is necessary to elucidate the pathogenic role of LILRA3 in various autoimmune diseases and the function of LILRA3 per se.

This study has some limitations. First, although the number of TAK patients included is relatively large for a single institution, this study is still limited by the sample size. The small sample size of our study made it difficult to draw a firm conclusion especially on the association of *LILRA3* rs103294 with vascular damage, because our study included only one patient with CC genotype of *LILRA3* rs103294. Further study with a larger sample size is awaited. Second, this study was based on a retrospective review of patients. Further prospective studies are required to confirm the findings of this study. Third, there might be a bias in the assessment and treatment of TAK, because this study was conducted in a single institution. Fourth, we did not assess disease activity measures such as Birmingham Vasculitis Activity Score and Indian Takayasu Clinical Activity Score. Fifth, since all the patients included in this study were Asian, mostly Japanese, our findings may not be applicable to patients with other ethnic backgrounds. Finally, vasculitis-associated damage measures (i.e. TADS and VDI) and angiographic scores (i.e. ASS, ADS, and ACS) were evaluated at different time points in each patient, because a recent whole-body contrast-enhanced angiography was not available in a substantial proportion of patients. This might have led to the discrepancy between the results on vasculitis-associated damage measures and angiographic scores.

In conclusion, our study showed for the first time that *IL12B* rs6871626 is associated with vascular damage in TAK, whereas *LILRA3* rs103294 was not. *IL12B* rs6871626 cannot be only a susceptibility marker, but also a marker for disease progression in TAK. Better understanding of the pathogenesis with the aid of genetic studies may enable physicians to fine-tune the treatment strategy for TAK based on the genotype of individual patients.

## Methods

### Patients

We enrolled 99 TAK patients who were enrolled in our previous genome-wide association study (GWAS)^5^ and whose genotypes for rs6871626, rs103294, and HLA-B*52 were available. The patients fulfilled either the 1990 American College of Rheumatology classification criteria^38^ or the diagnostic criteria for the Japan Circulation Society 2008^39^. This was a retrospective cohort study, and patient information in the electronic patient chart was retrospectively reviewed. We obtained approval from the Ethics Committee of Kyoto University Graduate School and the Faculty of Medicine for the study (No. G1006-9). Written informed consent was obtained from all patients. All study procedures were performed in accordance with the principles of the Declaration of Helsinki.

### Clinical evaluation

The presence or absence of AR, ischemic heart disease, cerebrovascular events, visual loss, renal replacement therapy, and inflammatory bowel disease were evaluated as organ involvement. The diagnosis of AR was based on transthoracic echocardiography and was used to detect AR. Ischemic heart disease was defined as angina pectoris or myocardial infarction requiring vascular interventions such as coronary artery bypass graft or percutaneous coronary intervention. Cerebrovascular events were included only if they had focal neurological signs such as paresis and weakness. Renal replacement therapy was defined as the condition requiring chronic hemodialysis. Inflammatory bowel disease was diagnosed after scrutiny by gastroenterologists, including endoscopic evaluation and cautious differential diagnosis, to exclude other causes such as drug-induced enterocolitis and infections.

### Arterial involvement

Involvement of individual arteries was comprehensively determined using digital subtraction angiography, enhanced computed tomography (CT), magnetic resonance angiography (MRA), ultrasonography, or fluorodeoxyglucose positron emission tomography to differentiate other conditions such as atherosclerosis.

To perform a systemic evaluation of arterial involvement in a qualitative and quantitative manner, we assessed ASS, ADS, and ACS^13^. These scores were calculated with the last whole-body contrast-enhanced CT angiography or MRA of each patient if available.

### Evaluation of vascular damage

Vasculitis-associated damage was evaluated using TADS^10^ and VDI^11^. TADS captures TAK-specific vascular damage, whereas VDI is applied to all types of systemic vasculitides^9,12^. TADS and VDI were measured at the last visit before November 2020.

### Treatments

We obtained clinical data regarding the administration of glucocorticoids, immunosuppressants, and biological disease-modifying anti-rheumatic drugs such as infliximab, tocilizumab, and ustekinumab.

### Vascular surgery and catheter intervention

The medical history of coronary artery bypass grafting, percutaneous coronary intervention, aortic valve replacement, aortic aneurysm repair, and bypass surgery were retrospectively reviewed.

### Genotyping

Illumina Infinium arrays were used for genome scanning, as previously described^5^. Genotypes of the HLA-B allele were identified by the Luminex microbead method in the NPO HLA laboratory (Kyoto, Japan).

### Statistical analysis

Data are presented as the mean ± standard deviation for continuous variables and numbers (%) for categorical variables.

We generally adopted the additive model to analyze the association between genotypes of SNPs and clinical characteristics. To determine the association between genotypes of SNPs and categorical variables, such as the frequency of organ involvement, arterial involvement, and treatment profiles, logistic regression analysis was performed to calculate the odds ratios (ORs) and 95% confidence intervals (CIs) of the risk allele. To determine the correlation between genotypes of SNPs and continuous variables, such as age, TADS, and VDI, linear regression analysis was performed to calculate the effect size (β) of the risk allele.

In addition, we also analyzed the association of *IL12B* rs6871626 with clinical characteristics using the dominant model. The Mann–Whitney test was used to compare continuous variables, and Fisher’s exact test was used to compare categorical variables*.* We did not use the dominant model in the analysis of *LILRA3* rs103294, because there was only one patient with CC genotype of *LILRA3* rs103294.

Statistical analyses were performed using JMP Pro 14.0.0 (SAS Institute, Cary, NC, USA), and p values < 0.05 were considered significant.

### Ethics approval and consent to participate

We obtained the approvals of the Ethics Committee of Kyoto University Graduate School and Faculty of Medicine for the study (No. G1006-9). Written informed consent was obtained from all patients. All study procedures were performed in accordance with the principles of the Declaration of Helsinki.

## Supplementary Information

Supplementary Legend.

Supplementary Figure.

Supplementary Table.

## Data Availability

The datasets used and analyzed during the current study are available from the corresponding author upon reasonable request.

## Abbreviations

AR: Aortic regurgitation
CI: Confidence interval
GWAS: Genome-wide association study
OR: Odds ratio
SNP: Single nucleotide polymorphism
TADS: Takayasu arteritis damage score
TAK: Takayasu arteritis
UST: Usutekinumab
VDI: Vasculitis Damage Index
