# Supplementary material for: A susceptibility locus in the IL12B but not LILRA3 region is associated with vascular damage in Takayasu arteritis
Source: Sci Rep. 2021 Jul 1;11:13667. doi: 10.1038/s41598-021-93213-9 (PMC8249518; doi:10.1038/s41598-021-93213-9)
Supplement: Supplementary file 1 — Supplementary Legend. [file 41598_2021_93213_MOESM1_ESM.docx]

Supplementary Figure Association of genotypes with systemic arteritis involvement on imaging.

The association of (A) *IL12B* rs6871626 and (B) *LILRA3* rs103294 with Arteritis Stenosis Score and Arteritis Dilation Score.
