# Supplementary figures and images for: A susceptibility locus in the IL12B but not LILRA3 region is associated with vascular damage in Takayasu arteritis
Source: Sci Rep. 2021 Jul 1;11:13667. doi: 10.1038/s41598-021-93213-9 (PMC8249518; doi:10.1038/s41598-021-93213-9)

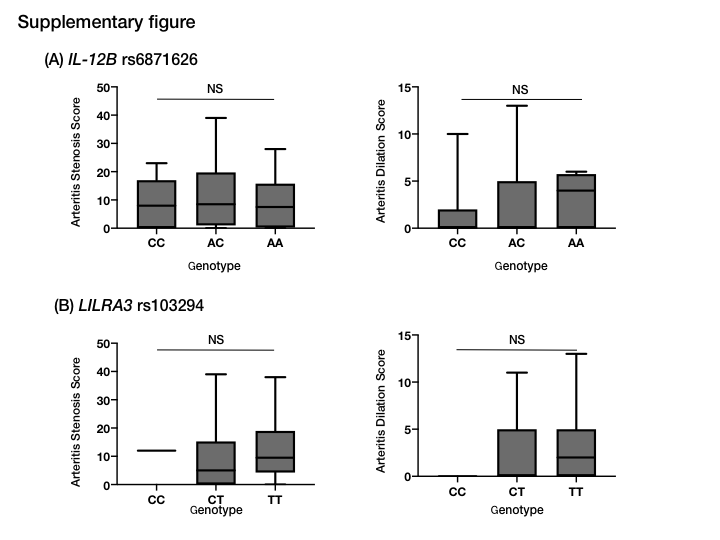

Supplement: Supplementary file 2 — Supplementary Figure. [file 41598_2021_93213_MOESM2_ESM.tiff]
