# Supplementary material for: A susceptibility locus in the IL12B but not LILRA3 region is associated with vascular damage in Takayasu arteritis
Source: Sci Rep. 2021 Jul 1;11:13667. doi: 10.1038/s41598-021-93213-9 (PMC8249518; doi:10.1038/s41598-021-93213-9)
Supplement: Supplementary file 3 — Supplementary Table. [file 41598_2021_93213_MOESM3_ESM.docx]

**Title**

A susceptibility locus in the *IL12B* but not *LILRA3* region is associated with vascular damage in Takayasu Arteritis

**Authors**

Keiichiro Kadoba^1^, Ryu Watanabe^2^*, Takeshi Iwasaki^1,3^, Toshiki Nakajima^4^, Koji Kitagori^1^, Shuji Akizuki^1^, Kosaku Murakami^1^, Ran Nakashima^1^, Motomu Hashimoto^2^, Masao Tanaka^2^, Koichiro Ohmura^1^, Akio Morinobu^1^, Chikashi Terao^1,5,6,7^, Hajime Yoshifuji^1^

1. Department of Rheumatology and Clinical Immunology, Graduate School of Medicine, Kyoto University, Kyoto, Japan.

2. Department of Advanced Medicine for Rheumatic Diseases, Graduate School of Medicine, Kyoto University, Kyoto, Japan.

3. Center for Genomic Medicine, Graduate School of Medicine, Kyoto University, Kyoto, Japan.

4. Department of Clinical Immunology and Rheumatology, the Tazuke-Kofukai Medical Research Institute, Kitano Hospital, Osaka, Japan.

5. Laboratory for Statistical and Translational Genetics, Center for Integrative Medical Sciences, RIKEN Center for Integrative Medical Sciences, Yokohama, Japan.

6. Clinical Research Center, Shizuoka General Hospital, Shizuoka, Japan.
7. The Department of Applied Genetics, The School of Pharmaceutical Sciences, University of Shizuoka, Shizuoka, Japan.

Supplementary table. Clinical characteristics of the subjects stratified by *IL12B* rs6871626 genotypes (the dominant model)

|  | *IL12B* rs6871626 | | P value |
| --- | --- | --- | --- |
|  | CC | AC/AA |  |
| Number | 19 | 80 |  |
| Background |  |  |  |
| Age at onset (years) | 33 ± 16 | 31 ± 14 | 0.82 |
| Female | 18 (95%) | 76 (95%) | 1.00 |
| Organ involvement |  |  |  |
| Visual loss | 0 (0%) | 3 (4%) | 1.00 |
| Hypertension | 10 (53%) | 44 (56%) | 0.80 |
| Aortic regurgitation | 8 (42%) | 53 (68%) | 0.62 |
| Ischemic heart disease | 1 (5%) | 5 (6%) | 1.00 |
| Cerebrovascular event | 0 (0%) | 9 (12%) | 0.20 |
| Renal replacement therapy | 0 (0%) | 1 (1%) | 1.00 |
| Inflammatory bowel disease | 1 (5%) | 6 (8%) | 1.00 |
| Ulcerative colitis | 1 (5%) | 4 (5%) | 1.00 |
| Crohn disease | 0 (0%) | 2 (3%) | 1.00 |
| Medication |  |  |  |
| Glucocorticoid | 15 (79%) | 64 (80%) | 1.00 |
| Oral immunosuppressant | 5 (36%) | 33 (42%) | 0.30 |
| Biologic | 1 (5%) | 16 (20%) | 0.18 |
| Intervention |  |  |  |
| Coronary artery bypass grafting | 2 (11%) | 3 (4%) | 0.25 |
| Percutaneous coronary intervention | 2 (11%) | 5 (6%) | 0.62 |
| Aortic valve replacement | 1 (5%) | 8 (10%) | 1.00 |
| Aneurysm repair | 2 (11%) | 8 (10%) | 1.00 |
| Bypass surgery | 2 (11%) | 7 (9%) | 1.00 |
| Vascular damage |  |  |  |
| Takayasu arteritis damage score | 3.4 ± 2.7 | 4.7 ± 3.2 | 0.11 |
| Vasculitis damage index | 3.5 ± 2.0 | 4.7 ± 2.4 | 0.06 |
| Data are n (%) or mean ± standard deviation. | | | |
